# Evaluation of the contribution of gut microbiome dysbiosis to cardiac surgery-associated acute kidney injury by comparative metagenome analysis

**DOI:** 10.3389/fmicb.2023.1119959

**Published:** 2023-03-17

**Authors:** Ying Li, Xinyi Jiang, Jingchun Chen, Yali Hu, Yunpeng Bai, Wang Xu, Linling He, Yirong Wang, Chunbo Chen, Jimei Chen

**Affiliations:** ^1^Department of Cardiac Surgery, Guangdong Cardiovascular Institute, Guangdong Provincial People's Hospital, Guangdong Academy of Medical Sciences, Guangzhou, China; ^2^Department of Intensive Care Unit of Cardiovascular Surgery, Guangdong Cardiovascular Institute, Guangdong Provincial People's Hospital, Guangdong Academy of Medical Sciences, Guangzhou, China; ^3^Department of Critical Care Medicine, Guangdong Provincial People's Hospital (Guangdong Academy of Medical Sciences), Southern Medical University, Guangzhou, China; ^4^Guangdong Provincial Key Laboratory of South China Structural Heart Disease, Guangzhou, China; ^5^School of Medicine, South China University of Technology, Guangzhou, China; ^6^School of Biology and Biological Engineering, South China University of Technology, Guangzhou, China; ^7^BGI College and Henan Institute of Medical and Pharmaceutical Sciences, Zhengzhou University, Zhengzhou, China; ^8^Center of Scientific Research, Maoming People’s Hospital, Maoming, China; ^9^The Second School of Clinical Medicine, Southern Medical University, Guangzhou, China; ^10^Shantou University Medical College, Shantou, China; ^11^Department of Emergency, Maoming People’s Hospital, Maoming, China

**Keywords:** acute kidney injury, gut microbiota dysbiosis, cardiac surgery, metagenomic sequencing, biomarker

## Abstract

**Introduction:**

Cardiac surgery-associated acute kidney injury (CSA-AKI) is a common hospital-acquired AKI that carries a grave disease burden. Recently, gut-kidney crosstalk has greatly changed our understanding of the pathogenesis of kidney diseases. However, the relationship between gut microbial dysbiosis and CSA-AKI remains unclear. The purpose of this study was to investigate the possible contributions of gut microbiota alterations in CSA-AKI patients.

**Methods:**

Patients undergoing cardiac surgery were enrolled and divided into acute kidney injury (AKI) and Non_AKI groups. Faecal samples were collected before the operation. Shotgun metagenomic sequencing was performed to identify the taxonomic composition of the intestinal microbiome. All groups were statistically compared with alpha- and beta-diversity analysis, and linear discriminant analysis effect size (LEfSe) analysis was performed.

**Results:**

A total of 70 individuals comprising 35 AKI and 35 Non_AKI were enrolled in the study. There was no significant difference between the AKI and Non_AKI groups with respect to the alpha-and beta-diversity of the Shannon index, Simpson or Chao1 index values except with respect to functional pathways (*p* < 0.05). However, the relative abundance of top 10 gut microbiota in CSA-AKI was different from the Non_AKI group. Interestingly, both LEfSe and multivariate analysis confirmed that the species *Escherichia coli*, *Rothia mucilaginosa*, and *Clostridium innocuum* were associated with CSA-AKI. Moreover, correlation heat map indicated that altered pathways and disrupted function could be attributed to disturbances of gut microbiota involving *Escherichia coli*.

**Conclusion:**

Dysbiosis of the intestinal microbiota in preoperative stool affects susceptibility to CSA-AKI, indicating the crucial role of key microbial players in the development of CSA-AKI. This work provides valuable knowledge for further study of the contribution of gut microbiota in CSA-AKI.

## Introduction

Acute kidney injury (AKI) is a clinically severe syndrome with a wide range of morbidity and mortality. Surveys have shown that AKI occurs in approximately 13.5–41.2% of ICU patients and sometimes exceeds 50%, with poor prognosis, such as prolonged hospital stay, high medical expenses and increased mortality (Deng et al., 2017; Yang et al., 2017; Ronco et al., 2019; Hu et al., 2022). For patients undergoing cardiac procedures, one of the most common life-threatening complications is cardiac surgery-associated AKI (CSA-AKI), ranging from 5 to 42% and even more (Helgason et al., 2021; Ostermann et al., 2021). Persistent renal malperfusion could lead to AKI ranging from mild renal dysfunction to loss of kidney function requiring renal replacement therapy (RRT) and even accelerate progression to chronic kidney disease (CKD). Timely recognition of CSA-AKI as soon as possible could be conducive to efficient administration of clinical care and appropriate intervention (Bai et al., 2022a).

The microbiota, known as “the hidden organ,” serves to conduct fermentation of food and defend against pathogens, and ensures mucosal immunity and intestinal environment homeostasis (Hou et al., 2022). Recently, advancements in metagenomic analysis have dramatically improved our understanding of the intestinal microbiota in maintaining a mutual symbiotic relationship with the host, and crosstalk between the gut and distant organs were descripted in several researches (Fang et al., 2022a,b; Wozniak et al., 2022; Zhang et al., 2022). A growing body of evidence indicates that the gut microbiota plays a significant role in various human diseases, including obesity, type 2 diabetes, atherosclerosis, myocardial infarction, hypertension, organ fibrosis, fatty liver and CKD (Jie et al., 2017; Marques et al., 2017; Loomba et al., 2019; McMillan and Hazen, 2019; Witkowski et al., 2020; Iatcu et al., 2021; Zaky et al., 2021; Zhu et al., 2021; Costa et al., 2022). Based on the gut-kidney axis, imbalance of the microbial community results in disruption of the intestinal epithelial barrier and accumulation of kidney toxins; in turn, kidney injury provokes systemic inflammation and oxidative stress to aggravate repercussions on the intestine. The translocation of bacterial products from the gut of CKD patients activates tissue macrophages and innate immunity, which could provide an explanation for the systemic inflammation that is associated with CKD and the end-stage renal disease (ESRD) (Anders et al., 2013). In addition, emerging data suggest that AKI is also affected by the intestinal environment (Gong J. et al., 2019; Kobayashi et al., 2021).

An increasing amount of data has highlighted the ability of gut microbiota to serve as screening, prognostic and predictive biomarkers in various diseases and the potential of modulating microorganisms to prevent diseases, augment therapies and restore intestinal health (Hiippala et al., 2018; Wong and Yu, 2019; Averina et al., 2020; Wei et al., 2021; Dong et al., 2022). However, the potential of the preoperative intestinal flora as a biomarker for CSA-AKI remains unknown. Thus, to explore the contribution of the preoperative phenotype of the gut microbiota to the development of CSA-AKI, we collected preoperative feces from patients undergoing cardiovascular operations and performed comparative metagenome-associated analyses on the intestinal microbial composition, which will provide a fresh perspective for the elucidation of the pathogenic mechanism of CSA-AKI and the discovery of potential biomarkers for ischemic AKI.

## Materials and methods

### Study design and population

In the current study, patients undergoing cardiac operations from two centers, Maoming People’s Hospital and Guangdong Provincial People’s Hospital, between November 2020 and August 2022 were enrolled to evaluate the association between preoperative intestinal flora composition and susceptibility to postoperative CSA-AKI. Fecal samples were collected before cardiac surgery. Reasons for exclusion were as follows: (1) age under 18 years; (2) received systemic antibiotic treatment within 48 h before admission; (3) a history of nephrectomy or ESRD or renal transplantation; (4) died within 24 h after cardiovascular surgery; (5) lack of preoperative fecal samples. Written informed consent was obtained from the patients before the surgery. The study was performed according to the guidelines in the Helsinki Declaration, and the local ethics committee approved the study with no. PJ2020MI-021-01 and no. KY-Q-2021-109-04.

Demographic and baseline variables, including sex, age, body mass index, history of diseases, and others, were recorded at admission to the hospital, as well as the perioperative clinical indicators. In addition, indicators of renal function, including blood urea nitrogen (BUN), serum creatinine (Scr) and cystatin C (Cys C), were also recorded. BUN-pre, Scr-pre, and Cys C-pre were defined as the preoperative values of BUN, Scr, and Cys C, respectively, while BUN-d1, Scr-d1, and Cys C-d1 were defined as the values of BUN, Scr, and Cys C on the first postoperative day. BUN-d2, Scr-d2, and Cys C-d2 represent the values of BUN, Scr, and Cys C on the second postoperative day, respectively. AKI was defined by the Kidney Disease Improving Global Outcomes (KDIGO) Clinical Practice Guidelines based on serum creatinine criteria (Khwaja, 2012). Finally, for the sake of comparability, a total of 70 patients comprising 35 AKI and 35 Non_AKI were enrolled.

### Shotgun metagenomic sequencing

Shotgun metagenomic sequencing was used for the analysis of the taxonomic composition of the intestinal microbiomes. Stool samples were collected before the cardiac surgery and immediately frozen at −80°C. MagPure Fast Stool DNA KF Kit B was used for the extraction of DNA, which was detected by DNA Oubit and AGE electrophoresis. All DNA samples were used to construct genomic libraries, including DNA fragmentation, end repair and addition of A-tail, adapter ligation, PCR amplification, single strand cyclization and library quality control. Library construction was performed on the automated system MGISP-960 using the MGIEasy DNA Library Preparation Kit. And the libraries were quantified using the Agilent 2100 Bioanalyzer. Then, paired-end metagenomic sequencing was performed on the DNBSEQ-T1 platform with a 350 bp insert size and 100 bp read length.

### Bioinformatic analysis

For the raw data, adaptor and low-quality reads (≤70) were discarded by the default mode of fastp, and the remaining reads were filtered to eliminate host DNA based on the human genome reference (hg38) using the “very sensitive” mode of bowtie2. On average, 6.96 Gb of high-quality nonhost sequences were obtained per sample in the research. HMP unified metabolic analysis network 3 (HUMAnN3) was performed to analyze high-quality reads in each sample to obtain the compositions of the microbial communities and determine the abundance of microbial pathways. Metagenomic phylogenetic analysis (MetaPhlAn3) embedded in the HUMAnN3 and Chocophlan pangenome databases was used to obtain species composition information, and the MinPath, Diamond and UniRef and MetaCyc databases were used to annotate gene families, functions and pathways. Finally, the HUMAnN3 algorithm generated gene-family abundance, pathway abundance and pathway coverage profiles. Moreover, abundance outputs has been normalized using the humann_renorm_table command.

The within-diversity of the sample and the intersample diversity were determined by alpha- and beta-diversity analyses using the Shannon, Simpson, and Chao1 indices and Bray–Curtis dissimilarity. Linear discriminant effect size analysis (LEfSe) was used to screen out differentially abundant taxa at the different levels. After identifying the biologically most characterized gut flora, log-linear discriminant analysis (LDA) scores were calculated. Differences between groups were also assessed using 2-tailed Welch’s *t*-tests with STAMP (v2.1.3).

### Statistical analyses

For clinical data, quantitative variables of non-normal distribution are presented as median and interquartile range (IQR) while variables of normal distribution are shown as mean and standard deviation. Vital signs were compared by paired *t*-tests or non-parametric tests according to the normality of variables. Categorical variables are expressed as percentage and compared by use of the Chi-square or Fisher’s test. Risk factors for CSA-AKI were investigated by logistic regression. First, a univariate analysis was performed and variables with a *p*-value <0.05 were included in the multivariate analysis. Shannon, Simpson, Chao1 index and Bray–Curtis dissimilarity were used for alpha diversity and beta diversity analyses. Permutational multivariate analysis of variance (PERMANOVA) was performed to assess whether the gut microbiota and metabolic pathway composition of different groups are significantly different. Spearman’s correlation was used for analysis of the relationship between intestinal flora and clinical data. A value of *p* < 0.05 was considered significant. R (v4.4.4) statistical software were used for above analyses.

## Results

### Demographic characteristics of the patients undergoing cardiac surgery

In the current study, 70 patients undergoing cardiac surgery between November 2020 and August 2022 were enrolled from two large medical centers in different regions in China (32 from Guangdong Provincial People’s Hospital and 38 from Maoming People’s Hospital). The basic information of the patients is shown in Table 1. The two groups exhibited no notable differences in most baseline characteristics (e.g., demographics, clinical history). Patients with CSA-AKI were older than the Non_AKI group. For cardiac surgery, both cardiopulmonary bypass time and operation time could increase the incidence of postoperative complications, which differ from other non-cardiac surgery. CSA-AKI patients in group had significantly elevated bilirubin and white blood cell (WBC) levels.

**Table 1:** Demographic and baseline characteristics of all patients.

Characteristics	Non_AKI (*n* = 35)	AKI (*n* = 35)	*P-* value
Age, years	53.1 (11.2)	60.0 (9.6)	0.008
Gender (male), n (%)	20 (51.7)	23 (65.0)	0.623
BMI, kg/m^2^	22.6 (2.8)	21.6 (3.5)	0.150
Admission Heart rate, times/min	80.0 (75.0–88.0)	80.0 (74.0–91.0)	0.737
Preexisting clinical conditions, n (%)			
Hypertension	10 (28.5)	12 (34.2)	0.607
Diabetes mellitus	2 (5.7)	2 (5.7)	1.000
Cerebrovascular disease	4 (11.4)	4 (11.4)	1.000
Coronary artery disease	3 (8.5)	5 (14.2)	0.710
Chronic kidney disease	1 (2.8)	2 (5.7)	1.000
ASA classification, n (%)			0.239
I ~ II	3 (8.6)	0 (0)	
III ~ IV	32 (91.4)	35 (100.0)	
Preoperative variables			
Serum creatinine, mg/dL	84.9 (67.4–96.8)	88.7 (75.6–113.0)	0.162
Urea nitrogen, mg/dL	6.3 (5.1–8.3)	6.5 (4.7–9.7)	0.953
Cystatin C, mg/L	0.9 (0.8–1.2)	1.1 (1.0–1.4)	0.011
Hemoglobin, g/L	131.5 (22.1)	116.1 (27.1)	0.012
WBC, ×10^9^/L	7.2 (5.7–9.2)	10.6 (6.8–15.7)	0.016
TBIL, μmol/L	13.7 (10.6–19.7)	23.9 (15.9–34.3)	0.006
Na^+^, mmol/L	138.2 (3.3)	139.9 (4.8)	0.089
K^+^, mmol/L	4.0 (0.4)	4.1 (0.7)	0.338
Intraoperative variables			
Cardiopulmonary bypass time, min	148.0 (120.0–205.0)	183.0 (150.0–245.0)	0.017
Operation time, min	300.0 (245.0–395.0)	360.0 (330.0–490.0)	0.013
Blood loss, mL	220.0 (200.0–300.0)	300.0 (200.0–300.0)	0.975
Fresh frozen plasma infusion, mL	0 (0-150)	0 (0)	0.773
Red blood cells infusion, U	1.6 (3.2)	1.0 (1.6)	0.470
Platelets infusion, U	0.2 (0.6)	0.1 (0.4)	0.723
APACHE II score	9.8 (3.2)	11.4 (4.5)	0.096

Results are presented as proportion for categorical variables, median (interquartile range)/mean (standard deviation) for continuous variables. BMI, body mass index; ASA, American Society of Anesthesiologists; WBC, White blood cell; TBIL, Total bilirubin; APACHE, Acute Physiology and Chronic Health Evaluation.

### Characteristics of gut microbiota in patients with CSA-AKI

**Figure 1:**
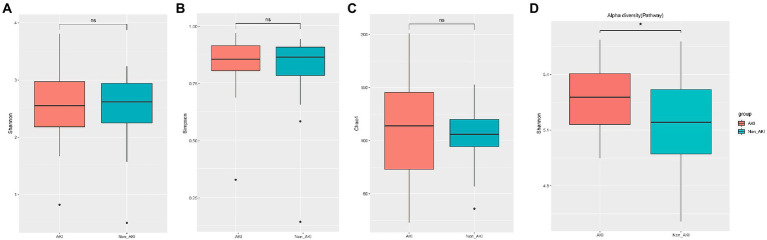
Comparison of gut microbiota α-diversities between the AKI and Non_AKI groups. Gut microbiota α-diversity according to Shannon index **(A)**, Simpson index **(B)**, Chao1 index **(C)** at the species level, and Shannon index at the functional pathway level **(D)**. Threshold for statistical significance: *p* = 0.05. * means *P* < 0.05.

**Figure 2:**
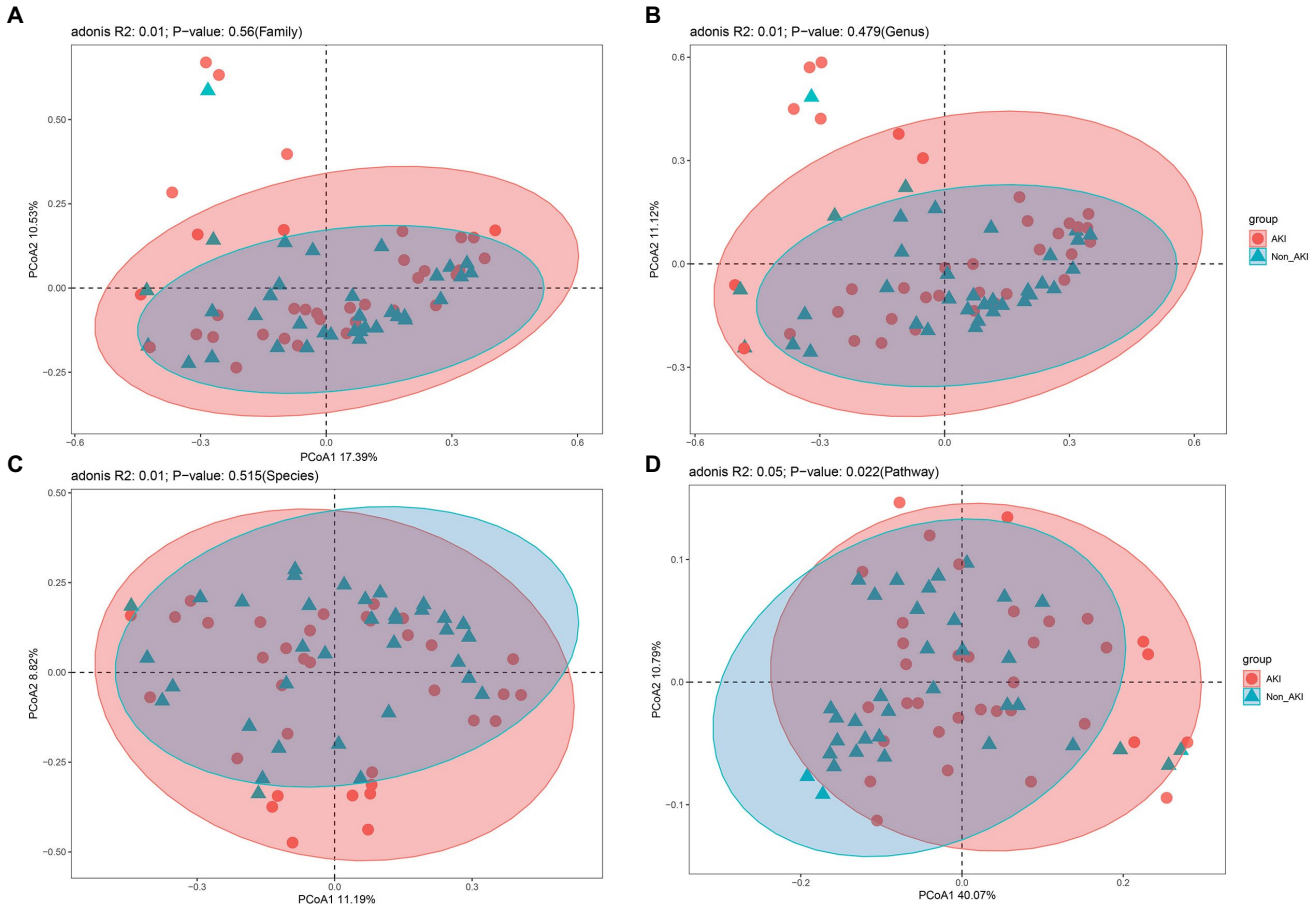
Comparison of beta-diversities using PCoA between the Non_AKI and AKI groups. **(A)** at the family level, **(B)** at the genus level, **(C)** at the species level, **(D)** at the pathway level. Threshold for statistical significance: *p* = 0.05.

Metagenomic sequencing of preoperative stool samples was performed to identify the association between gut microbiota and CSA-AKI. A total of 72 gut microbiota at the family level, 189 at the genus level and 604 at the species level were identified in 70 fecal samples after the removal of unannotated microbiota. To assess the overall diversity in microbial compositions and functions among the two groups, we performed alpha-diversity analysis by Shannon, Simpson, and Chao1 index (Figure 1) and beta-diversity analysis using PCoA (Figure 2) at the levels of family, genus, species and pathway. There was no significant difference between the AKI and Non_AKI groups with respect to the alpha diversity of the Shannon index at the family level (Supplementary Figure S1A), genus level (Supplementary Figure S1B), or species level (Figure 1A). Although the alpha-diversity of the Simpson and Chao1 indices in AKI was higher than that in Non_AKI to varying degrees, there was no significant difference (*p* > 0.05), suggesting similar preoperative gut microbiota diversity in patients undergoing CSA-AKI. However, the alpha-diversity of intestinal flora functional pathways in the AKI group was significantly higher than that in the Non_AKI group (*p* < 0.05) (Figure 1D). Based on beta-diversity analysis of PcoA, it seems that there were no significant differences at any levels between the two groups (Figures 2A–C) except for the pathway level (*p* < 0.05) (Figure 2D).

### Differences in the relative abundance of the top 10 gut microbiota in the CSA-AKI and Non_AKI groups

**Figure 3:**
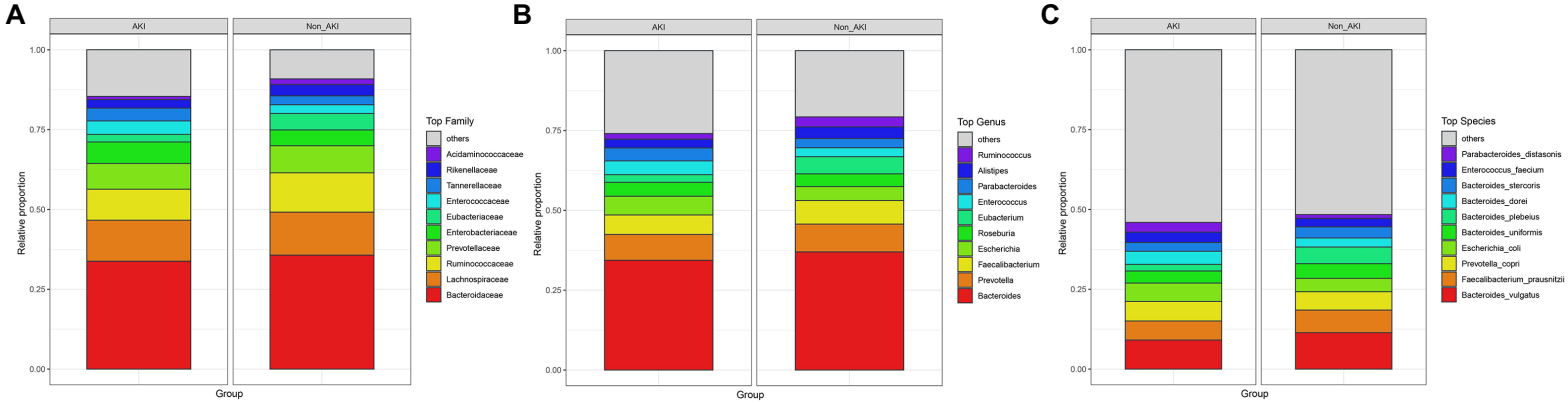
Top 10 relative abundance histograms at the family, genus, and species levels of the groups. **(A)** Top 10 relative abundance histograms at the family level of the groups. **(B)** Top 10 relative abundance histograms at the genus level of the groups. **(C)** Top 10 relative abundance histograms at the species level of the groups. The others represent the sums of the relative abundances of all others except the top 10.

We further analyzed the differences in gut microbiota composition between the AKI and Non_AKI groups. The top 10 gut microbiota with the highest abundance at the taxonomic levels were selected to generate an accumulation histogram of relative abundance (Figure 3 and Supplementary Table S1). The proportion of the top 10 varied greatly at different levels, in which the top 10 genera and species accounted for greater than 50% of the total. At the family level, *Bacteroidaceae*, *Lachnospiraceae*, and *Ruminococcaceae* had the highest abundance, while the proportions of *Enterobacteriaceae*, *Enterococcaceae*, and *Tannerellaceae* in the AKI group were much higher than those in the Non_AKI group (Figure 3A). For the top 10 genera and species, *Bacteroides vulgatus*, *Faecalibacterium prausnitzii*, and *Prevotella copri* belonged to *Bacteroides*, *Prevotella*, and *Faecalibacterium,* respectively, which accounted for the greatest abundance in the Non_AKI group. Figure 3B shows that *Escherichia*, *Enterococcus*, and *Parabacteroides* were significantly enriched in the AKI group. Compared with the Non_AKI group, in addition to *Escherichia coli* and *Parabacteroides distasonis*, *Bacteroides dynorei* was also much more prevalent in the AKI group at the species level, while other species belonging to *Bacteroides* were significantly enriched in the Non_AKI group (Figure 3C).

### Identification of bacterial species associated with CSA-AKI

**Figure 4:**
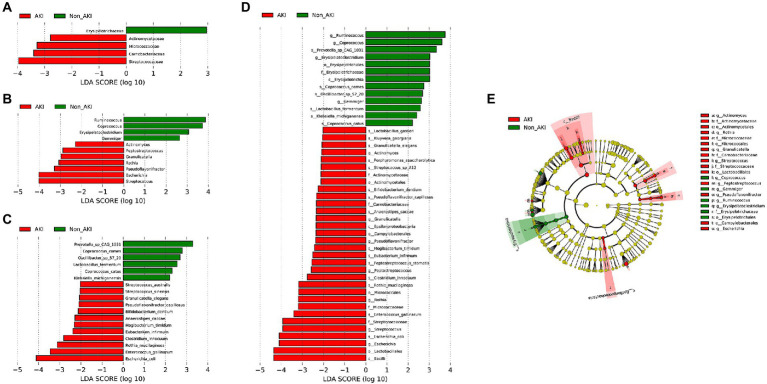
Variations in fecal microbiota composition determined by linear discriminant analysis (LDA) effect size analysis, LDA > 2.0, *p* < 0.05. AKI vs. Non_AKI at the family level **(A)**, the genus level **(B)**, the species level **(C)**, and the overall level **(D)**. **(E)** Taxonomic cladogram obtained from LEfSe analysis.

LEfSe analysis was further conducted to confirm the significant differences in fecal microbiota compositions at the family, genus and species levels between the AKI and Non_AKI groups, which are displayed in Figure 4. Compared to the Non_AKI group, the increased abundance of bacteria in the AKI group at the family level was enriched in *Streptococcaceae*, *Carnobacteriaceae*, *Micrococcaceae*, and *Actinomycetaceae*, whereas the decreased bacterial abundance was enriched in *Erysipelotrichaceae* (Figure 4A). At the genus level, the abundances of *Streptococcus*, *Escherichia*, *Pseudoflavonifractor*, *Rothia*, *Granulicatella*, *Peptostreptococcus*, and *Actinomyces* were significantly increased in the AKI group, while those of *Gemmiger*, *Erysipelatoclostridium*, *Coprococcus*, and *Ruminococcus* were decreased (Figure 4B). More specifically, the species of *Escherichia coli*, *Enterococcus gallinarum*, *Rothia mucilaginosa*, and *Clostridium innocuum* were found to be significantly more enriched in the AKI group, whereas Non_AKI patients exhibited greater abundance of *Prevotella sp. CAG: 1031, Coprococcus comes, Oscillibacter sp. 57_20*, and *Lactobacillus fermentum* (Figure 4C). Overall, LEfSe revealed significant differences between the two groups: in particular, Figures 4D,E show that the class *Bacilli*, genus *Escherichia*, species *Escherichia coil*, and genus *Streptococcus* were more enriched in the AKI group, while the genera *Ruminococcus*, *Coprococcus*, *Prevotella sp. CAG: 1031*, and *Erysipelatoclostridium* were much more enriched in the Non_AKI group.

In addition, we assessed the contribution of four differential species to the development of CSA-AKI. After univariable analysis, age, operation time, WBC, hemoglobin, *Escherichia coli*, *Rothia mucilaginosa*, *Clostridium innocuum* were associated with CSA-AKI. After multivariate analysis, both *Escherichia coli*, *Rothia mucilaginosa*, and *Clostridium innocuum* remain independently associated with CSA-AKI (respectively OR: 0.549, 95%CI [0.316–0.953], *p* = 0.033; OR: 5.054, 95%CI [1.412–18.082], *p* = 0.013; OR: 3.356, 95%CI [1.278–8.810], *p* = 0.014) (Table 2).

**Table 2:** Factors associated with CSA-AKI.

Variables	Univariate analysis OR	95% CI	*P-*values
Age, years	1.068	1.041–1.125	0.012
Gender (male)	1.438	0.547–3.781	0.462
BMI, kg/m^2^	0.893	0.765–1.043	0.152
Admission Heart rate, times/min	1.010	0.980–1.041	0.519
Hypertension	1.304	0.474–3.590	0.607
Diabetes mellitus	1.000	0.133–7.527	1.000
Cerebrovascular disease	1.000	0.229–4.361	1.000
Coronary artery disease	1.778	0.391–8.092	0.457
Chronic kidney disease	2.061	0.178–23.826	0.563
APACHE II score	1.115	0.979–1.270	0.102
Cardiopulmonary bypass time, min	1.007	1.000–1.014	0.061
Operation time, min	1.004	1.000–1.008	0.038
WBC	1.157	1.031–1.299	0.014
Hemoglobin, g/L	0.974	0.954–0.995	0.016
Blood loss, mL	0.999	0.007–1.001	0.422
Fresh frozen plasma infusion, mL	1.000	0.998–1.002	0.724
Red blood cells infusion, U	0.882	0.711–1.094	0.254
Platelets infusion, U	0.797	0.308–2.063	0.640
Serum creatinine, mg/dL	1.003	0.996–1.011	0.351
Urea nitrogen, mg/dL	1.051	0.950–1.163	0.334
Cystatin C, mg/L	2.276	0.855–6.056	0.100
TBIL, μmol/L	1.033	0.995–1.072	0.092
Na^+^, mmol/L	1.115	0.980–1.267	0.098
K^+^, mmol/L	1.523	0.649–3.576	0.334
*Escherichia coli*	0.558	0.382–0.814	0.002
*Enterococcus gallinarum*	2.485	0.909–6.794	0.076
*Rothia mucilaginosa*	2.731	1.237–6.032	0.013
*Clostridium innocuum*	1.833	1.084–3.101	0.024
Variables	Multivariate analysis OR	95% CI	*P-*value
Age, years	1.088	1.004–1.180	0.040
WBC, ×10^9^/L	1.194	1.047–1.362	0.008
Hemoglobin, g/L	0.972	0.941–1.004	0.091
Operation time, min	1.006	0.999–1.013	0.077
*Escherichia coli*	0.549	0.316–0.953	0.033
*Rothia mucilaginosa*	5.054	1.412–18.082	0.013
*Clostridium innocuum*	3.356	1.278–8.810	0.014

BMI, body mass index; APACHE, Acute Physiology and Chronic Health Evaluation; WBC, White blood cell; TBIL, Total bilirubin, OR, odds ratio; 95%CI, 95% confidence interval.

### Gut microbial functional disruption in patients with CSA-AKI

**Figure 5:**
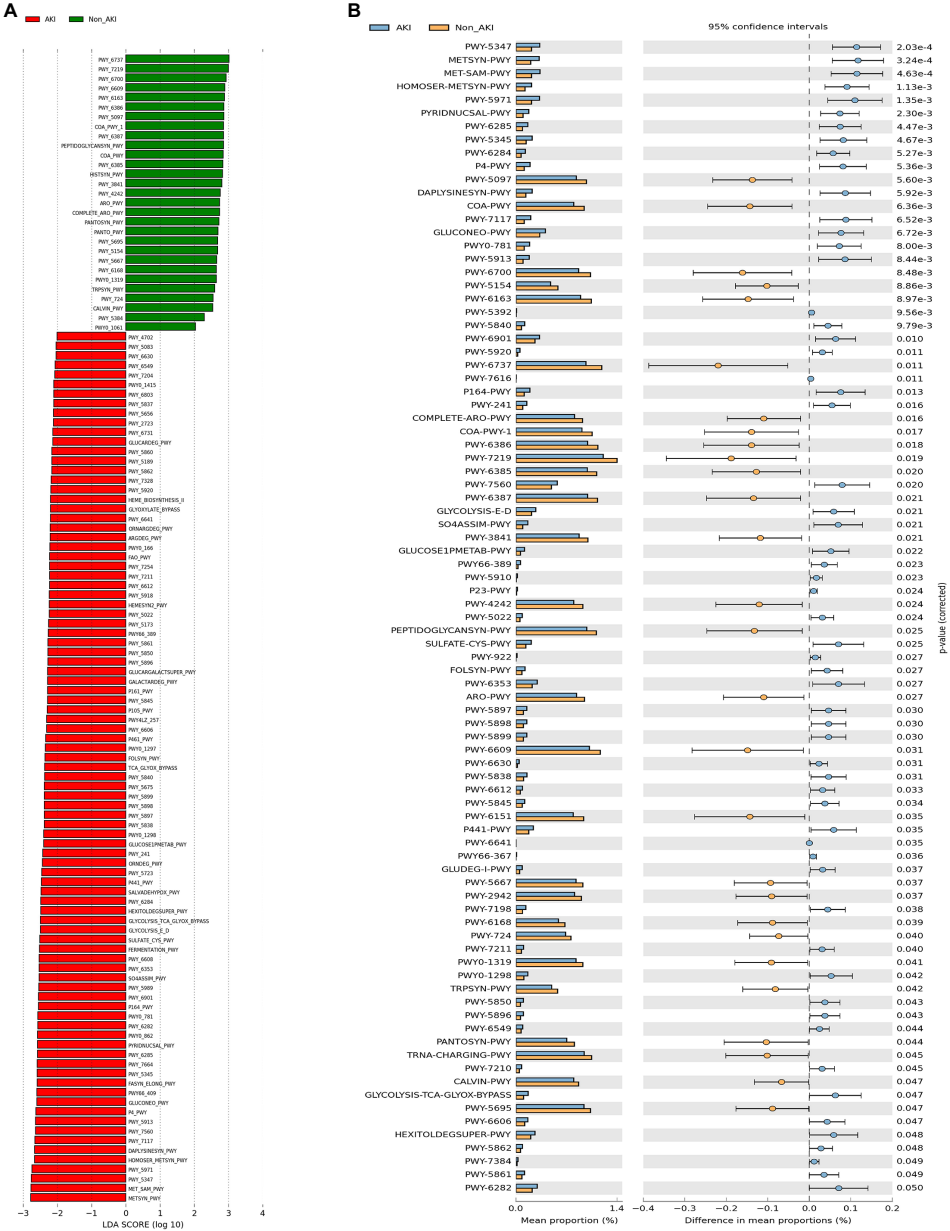
Pathway differences between the AKI and Non_AKI groups. **(A)** LEfSe analysis (LDA > 2); **(B)** STAMP analysis.

To compare the functional potential of the intestinal microbiota in preoperative stools in patients, we further investigated the pathway differences between the two groups by both LEfSe and STAMP analysis (Figure 5 and Supplementary Tables S2, S3). In general, a total of 91 pathways in AKI and 29 in the Non_AKI group were identified according to the LEfSe analysis (LDA > 2). There was primarily enrichment of L-homoserine and L-methionine biosynthesis, S-adenosyl-L-methionine biosynthesis, palmitate biosynthesis, L-lysine biosynthesis I, C4 photosynthetic carbon assimilation cycle, methylerythritol phosphate pathway II, and partial TCA cycle in the AKI group (LDA > 2.6), while starch degradation V, adenosine ribonucleotides *de novo* biosynthesis, queuosine biosynthesis I (*de novo*), adenine and adenosine salvage III, and chorismate biosynthesis from 3-dehydroquinate were enriched in the Non_AKI group (LDA > 2.8) (Figure 5A). Similarly, the STAMP results showed that 87 differential pathways were detected, 59 of which were in the AKI group, which was slightly different from the former analysis with variable proportions (Figure 5B). The pathways of adenosine ribonucleotides *de novo* biosynthesis (1.19 ± 0.30% vs. 1.38 ± 0.34% in the AKI and Non_AKI group, respectively, *p* = 0.019), starch degradation V (0.95 ± 0.35% vs. 1.17 ± 0.34%, *p* = 0.011), adenine and adenosine salvage III (1.00 ± 0.25% vs. 1.15 ± 0.30%, *p* = 0.030), UDP-N-acetylmuramoyl-pentapeptide biosynthesis II (lysine-containing) (0.98 ± 0.24% vs. 1.12 ± 0.23%, *p* = 0.018), methylerythritol phosphate pathway II (0.56 ± 0.13% vs. 0.49 ± 0.14%, *p* = 0.020), gluconeogenesis I (0.40 ± 0.10% vs. 0.33 ± 0.13%, *p* = 0.007), S-adenosyl-L-methionine biosynthesis (0.33 ± 0.14% vs. 0.21 ± 0.12%, *p* < 0.001), and L-methionine biosynthesis (transsulfuration) (0.33 ± 0.12% vs. 0.21 ± 0.12%, *p* < 0.001) were significantly different between the AKI and Non_AKI groups.

**Figure 6:**
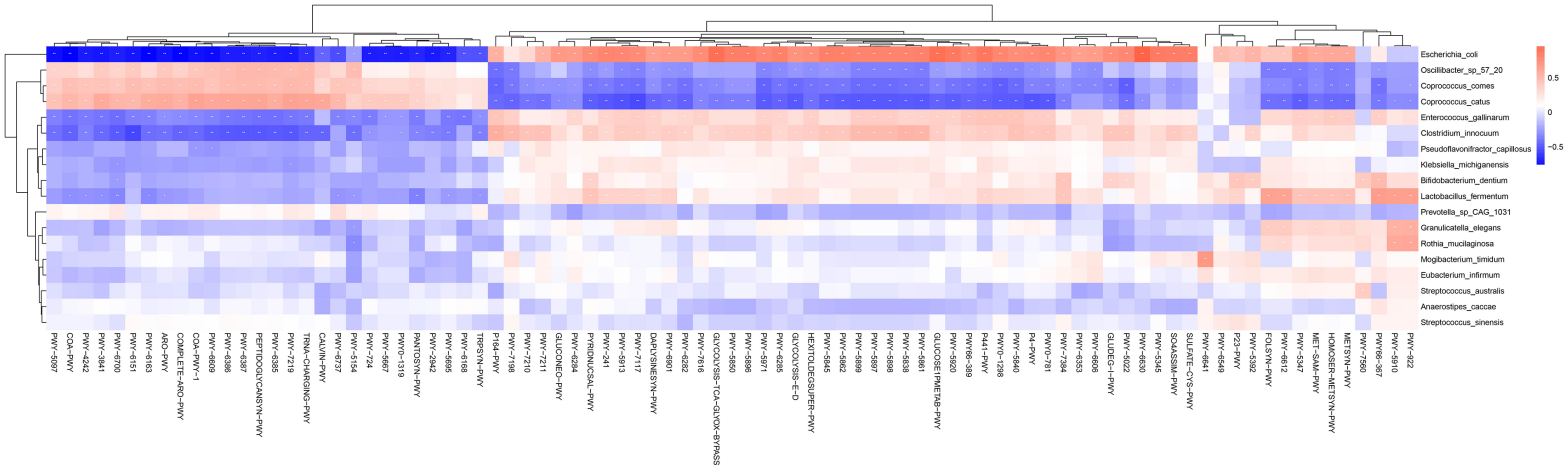
Heatmap showing the association between the differential intestinal microbiota of species and 87 differential pathways (STAMP analysis) in all patients. Single asterisks indicate statistical significance based on Spearman correlation with *p* < 0.05, and double asterisks indicate statistical significance with *p* < 0.01. Cor. coef., correlation coefficient.

Furthermore, analysis of the relationship between differential intestinal flora and differential functional pathways was conducted based on the results of STAMP (Figure 6 and Supplementary Table S3) and LEfSe (Supplementary Figure S2). The correlation heatmap indicated that altered pathways and disrupted function could be attributed to disturbances of gut microbiota involving *Escherichia coli*, *Oscillibacter sp. 57_20*, *Corprococcus comes*, *Coprococcus catus*, *Enterococcus gallinarum*, *Clostridium innocuum*, *Lactobacillus fermentum*, and others.

### Correlation between gut microbiota of metagenomics data and clinical parameters of AKI

**Figure 7:**
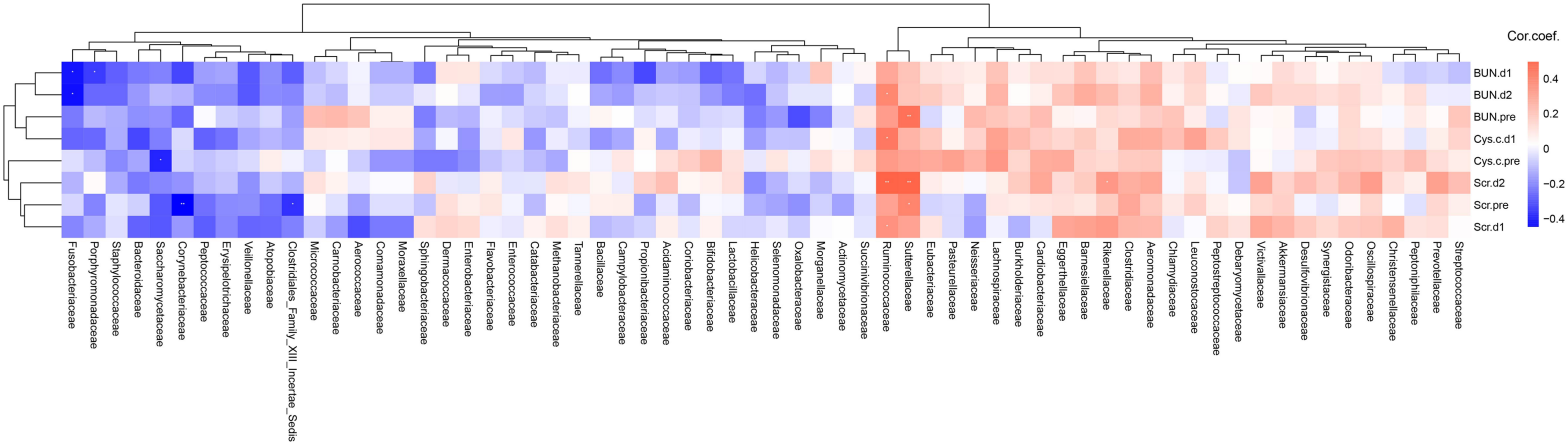
Heatmap showing the association between the eight clinical renal functional variables and family abundance in the gut microbiome of CSA-AKI patients. Single asterisks indicate statistical significance based on Spearman correlation with *p* < 0.05, and double asterisks indicate statistical significance with *p* < 0.01. Cor. coef., correlation coefficient; BUN-pre, preoperative blood urea nitrogen; BUN-d1, blood urea nitrogen on postoperative day 1; BUN-d2, blood urea nitrogen on postoperative day 2; Scr-pre, preoperative blood urea nitrogen; Scr-d1, blood urea nitrogen on postoperative day 1; Scr-d2, blood urea nitrogen on postoperative day 2; Cys C-pre, preoperative blood urea nitrogen; Cys C-d1, blood urea nitrogen on postoperative day 1.

Correlation analysis between the gut microbiome and clinical renal function parameters, including blood urea nitrogen, serum creatinine, and cystatin C, was performed to elucidate the roles of different levels of gut microbiota in patients with CSA-AKI. Interestingly, we discovered a significant positive correlation between *Sutterellaceae* and BUN-pre (*p* < 0.01) and Scr-pre (*p* < 0.01) at the family level, while *Ruminococcaceae* was positively correlated with postoperative indices, such as BUN-d2, Cys C-d1, Scr-d2, and Scr-d1 (Figure 7). Moreover, *Saccharomycetaceae, Corynebacteriaceae,* and *Clostridiales Family XIII. Incertae Sedis* were negatively correlated with preoperative renal function indices involving Cys-C-pre and Scr-pre. At the species level (Supplementary Figure S3), *Sutterella parvirubra* was positively correlated with BUN-pre (*p* < 0.01), Scr-pre (*p* < 0.05), Cys C-pre (*p* < 0.05), and Scr-d2 (*p* < 0.01). However, *Clostridium perfringens* was negatively correlated with BUN-pre (*p* < 0.05), Scr-pre (*p* < 0.05), and Scr-d2 (*p* < 0.05). It seems that *Ruminococcaceae bacterium D16 and Parabacteroides sp. CAG:409* were mainly positively correlated with those three renal function indicators 24 h after the operation, while *Butyricicoccus pullicaecorum* was negatively correlated with those indicators 24–48 h after the operation.

## Discussion

Herein, based on metagenomic analysis, the study found that CSA-AKI was significantly affected by the preoperative gut microbiota, particularly *Escherichia coli*, *Enterococcus gallinarum*, and *Rothia mucilaginosa*, indicating the pivotal role of the gut microbiota in the development of CSA-AKI. To the best of our knowledge, this research explored the potential of gut microbiota in the early identification of kidney injury and provided the first direct evidence that the preoperative phenotype of gut microbiota could affect susceptibility to CSA-AKI, which paves the way to clinically translate the use of gut microbiota for CSA-AKI in the near future.

The gut microbiota is considered a dynamic organ that maintains constant communication and symbiosis with the host and mediates susceptibility to various diseases (Gong S. et al., 2019; Fang et al., 2022b; Wozniak et al., 2022; Zhang et al., 2022). Firmicutes and Bacteroidetes predominate and constitute 90% of the gut microbiota, followed by Actinobacteria, Proteobacteria, and Verrucomicrobia (Arumugam et al., 2011; Rinninella et al., 2019; Wozniak et al., 2022). *Bacteroides* was the most abundant but variable genus in the study, which is consistent with a previous report (Arumugam et al., 2011). *Bacteroides vulgatus* was enriched both in the AKI and Non_AKI groups, while *Bacteroides uniformis*, *Bacteroides plebeius*, and *Bacteroides stercoris, with the exception of Bacteroides dynorei*, displayed higher abundance in the latter group. *Bacteroides vulgatus* and *Bacteroides dynorei* were reported to have significantly lower abundance in patients with coronary artery disease and attenuated atherosclerotic lesions and suppressed inflammation in mice (Yoshida et al., 2018). In metastatic melanoma patients, a high abundance of *Bacteroides dorei* seems to increase the risk of immune-related adverse events and is associated with enzyme capacity for adenosine metabolism, while *Bacteroides vulgatus* was dominant in the low-risk cluster (Usyk et al., 2021). These results show that *Bacteroides dorei* clustered in CSA-AKI patients could be harmful to the kidneys, which is in line with the aforementioned research. Further investigation into the intervention and regulation of targeted microbiota, including monocolonization, would be more conducive to explaining this finding.

Both LEfSe and multivariate analysis showed *Escherichia coli*, *Rothia mucilaginosa*, and *Clostridium innocuum* were independently associated with CSA-AKI, indicating that preoperative characteristics of fecal microbiota could affect susceptibility to CSA-AKI. *Escherichia coli*, a Gram-negative, is the predominant aerobic organism in the gut and is characteristic of both commensal and pathogenic bacteria (Tenaillon et al., 2010). Commensal *Escherichia coli* located in the large intestine, especially in the caecum and colon, and alteration of the commensal niche cause commensal strains to evolve into a pathogenic state. In the cohort of patients undergoing cardiovascular surgery, compared with the Non_AKI patients, *Escherichia coli* was significantly increased in the preoperative gut microbiota of CSA-AKI patients at the species level, indicating its key microbial role in kidney injury and the potential susceptibility to CSA-AKI. In fact, various species of *Escherichia coli* were reported to cause AKI and permanent renal failure (Maddens et al., 2012; Trachtman et al., 2012; Derad et al., 2016; Wang et al., 2020). *Samanta* et al. also found that AKI can occur under hypobaric hypoxia and affect the gut microbial populations of *Escherichia coli, Bacteroidetes*, *Bifidobacterium*, and *Salmonella* (Samanta et al., 2018). These results reinforced the understanding of the interaction between the gut microbiota of *Escherichia coli* and kidney injury. Notably, aromatic amino acids, including tryptophan and phenylalanine, which are associated with AKI (Piedrafita et al., 2021; Bai et al., 2022b; Nadour et al., 2022), could be further metabolized by *Escherichia coli* to mediate host-microbiome crosstalk (Liu et al., 2020). *Rothia mucilaginosa,* belonging to the family Micrococcaceae, is increasingly recognized as an opportunistic pathogen mostly affecting immunocompromised hosts. Interestingly, although it is considered a part of the normal microflora of the human mouth and the upper respiratory tract, the species was rarely reported in ESRD patients with heart transplant and exhibited potential as a microbial biomarker for necrotizing enterocolitis (Bejjanki and Koratala, 2019; Liu et al., 2022). To the best of our knowledge, *Escherichia coli* and *Rothia mucilaginosa* were first identified as differential species in preoperative stools from CSA-AKI patients in this study, and its potential as a biomarker still needs to be further explored.

In addition, *Enterococcus gallinarum* were significantly increased in CSA-AKI patients, while *Prevotella sp. CAG: 1031* was enriched in the Non_AKI group (LDA > 3), which differs from previous studies (Nakade et al., 2018; Andrianova et al., 2020; Yang et al., 2020). The main reason for the difference is that most of these studies were performed with mouse feces after surgery, whereas ours was performed with human feces before surgery. *Enterococcus gallinarum* is a Gram-positive facultative anaerobic bacterium with the capacity to cause nosocomial bloodstream infections (Mastor et al., 2020). Among long-term dialysis patients, *Enterococcus gallinarum* was reported to be a common vancomycin-resistant Enterococcus, accounting for 57.1% (Barbosa et al., 2004). Moreover, under the regulation of theabrownin, the reproduction of *Prevotella sp. CAG: 1031* belonging to Bacteroides was reported to help to reduce body weight and blood sugar levels, which seems to agree with our research and provides a basis for its beneficial function, even though no studies have validated the benign effect of *Prevotella sp. CAG: 1031* on kidney disease.

Concomitant with the alteration of gut microbial composition, we discovered dysbiosis in bacterial pathway functions. Superpathways of L-homoserine and L-methionine biosynthesis predominated in the AKI group, while pathways of starch degradation V and adenosine ribonucleotides *de novo* biosynthesis were substantially enriched in the Non_AKI group. In fact, L-methionine, an essential amino acid, is closely related to the metabolism of sulfur-containing compounds in organisms, and L-homoserine is an intermediate in the biosynthesis of L-methionine. Restriction of sulfur-containing amino acid intake seems to be instrumental in kidney protection and relief of ischemia/reperfusion injury (Pan et al., 2020; Osterholt et al., 2022). More specifically, *Escherichia coli*, *Oscillibacter sp. 57_20*, *Corprococcus comes*, and *Enterococcus gallinarum* are likely to make great contributions to the altered pathways and disrupted function. Furthermore, correlation analysis showed that *Butyricicoccus pullicaecorum*, a butyrate-producing bacterium, was negatively correlated with postoperative renal injury indicators, suggesting that decreased production of butyrate may be closely related to the occurrence of CSA-AKI. In fact, *Butyricicoccus pullicaecorum* attenuates colitis in rats and strengthens epithelial barrier function and is even regarded as a promising probiotic candidate for people suffering from inflammatory bowel disease (Eeckhaut et al., 2013; Geirnaert et al., 2014).

The main limitation of this study is the relatively small sample size, which can only represent the changes in fecal microbiota in a small proportion of CSA-AKI patients. A large sample size is required to verify the present results. Another limitation is the lack of causality demonstration. Although a differential gut microbiota was identified between the AKI and Non_AKI groups, the correlation does not imply causation. The study shows that the preoperative characteristics of the fecal microbiota could affect susceptibility to CSA-AKI, which to a certain extent implies that intestinal flora disturbance is one of the important factors for the occurrence of AKI. However, whether this is the effect of single or multiple bacterial species and how the flora exerts the effect still need to be further verified.

## Conclusion

Taken together, based on the metagenomic sequencing analysis of preoperative feces, the study herein identified differential microbiota between CSA-AKI and Non_AKI patients and revealed that preoperative characteristics of fecal microbiota could affect susceptibility to CSA-AKI, which deepens our understanding of the gut-kidney axis and pathogenesis of CSA-AKI. Meanwhile, these findings provide significant evidence for the potential role of gut microbiota as a key player in CSA-AKI, which will assist in future study design to accurately assess the potential biomarkers of gut microbiota and develop new targeted therapies for CSA-AKI.

## Data availability statement

The datasets presented in this study can be found in online repositories. The names of the repository/repositories and accession number(s) can be found below: https://ngdc.cncb.ac.cn/gsa/, PRJCA013107.

## Ethics statement

The studies involving human participants were reviewed and approved by the Medical Ethics Committee of Maoming People’s Hospital and Medical Ethics Committee of Guangdong Provincial People’s Hospital. The patients/participants provided their written informed consent to participate in this study.

## Author contributions

YL, XJ, and JCC programmed the task and coordinated the study. YL drafted the manuscript. YL and YH analyzed the data. YB and WX collected the fecal samples and clinical data. LH and YW participated in the methodology. CC and JMC provided financial support, supervision for the study. All authors have read and agreed to the published version of the manuscript.

## Funding

This research was funded by the National Nature Science Foundation of China (No. 82172162), the Major Program of Summit Project, Guangdong Province High-Level Hospital Construction Project of Guangdong Provincial People’s Hospital, Guangdong Academy of Medical Sciences (No. DFJH2020028), the Office of Talent Work Leading Team in Maoming (No. MaoRenCaiBan [2020]24, 200221115835503), the Science and Technology Planning Project of Guangdong Province (No. 2020B1111170011), and the Science and Technology Program of Guangzhou, China (No. 202206010049).

## Conflict of interest

The authors declare that the research was conducted in the absence of any commercial or financial relationships that could be construed as a potential conflict of interest.

## Publisher’s note

All claims expressed in this article are solely those of the authors and do not necessarily represent those of their affiliated organizations, or those of the publisher, the editors and the reviewers. Any product that may be evaluated in this article, or claim that may be made by its manufacturer, is not guaranteed or endorsed by the publisher.
