# Supplementary material for: Evaluation of the contribution of gut microbiome dysbiosis to cardiac surgery-associated acute kidney injury by comparative metagenome analysis
Source: Front Microbiol. 2023 Mar 17;14:1119959. doi: 10.3389/fmicb.2023.1119959 (PMC10091463; doi:10.3389/fmicb.2023.1119959)
Supplement: Supplementary file 1 [file Data_Sheet_1.pdf]

Supplementary Material

Evaluation of the contribution of gut microbiome dysbiosis to Cardiac Surgery-associated Acute Kidney Injury by Comparative Metagenome Analysis

Ying Li[1,2,9,10†], Xinyi Jiang[3,9†], Jingchun Chen[4,9†], Yali Hu[5], Yunpeng Bai[6], Wang Xu[2,7,] Linling He[8,9], Yirong Wang[2,4], Chunbo Chen[2] [illegible] Jimei Chen[1,10*]

[1]Department of Cardiac Surgery, Guangdong Cardiovascular Institute, Guangdong Provincial People's Hospital, Guangdong Academy of Medical Sciences, Guangzhou, 510080, China

[2]Department of Intensive Care Unit of Cardiovascular Surgery, Guangdong Cardiovascular Institute, Guangdong Provincial People's Hospital, Guangdong Academy of Medical Sciences, Guangzhou, 510080, China

[3]School of Medicine, South China University of Technology, Guangzhou, 510006, China

[4]School of Biology and Biological Engineering, South China University of Technology, Guangzhou, 510006, China

[5]BGI College & Henan Institute of Medical and Pharmaceutical Sciences, Zhengzhou University, Zhengzhou, 450052, China

[6]Center of Scientific Research, Maoming People's Hospital, Maoming, 525000, China

[7]The Second School of Clinical Medicine, Southern Medical University, Guangzhou, 510515, China

[8]Shantou University Medical College, Shantou, 515000, China

[9]Department of Critical Care Medicine, Guangdong Provincial People's Hospital (Guangdong Academy of Medical Sciences), Southern Medical University, Guangzhou, 510080, China

[10]Guangdong Provincial Key Laboratory of South China Structural Heart Disease, Guangzhou, 510080, China

***Correspondence:** *Chunbo Chen (email: gghccm@163.com),* Jimei Chen *jimei_1965@outlook.com)*

[†]These authors have contributed equally to this work.

1 Supplementary Figures

Figure S1. Comparison of gut microbiota α-diversities between the AKI and Non_AKI group. Gut bacteriobiota α-diversity according to Shannon index at the family (A), genus level (B). Threshold for statistical significance: $P = 0.05$

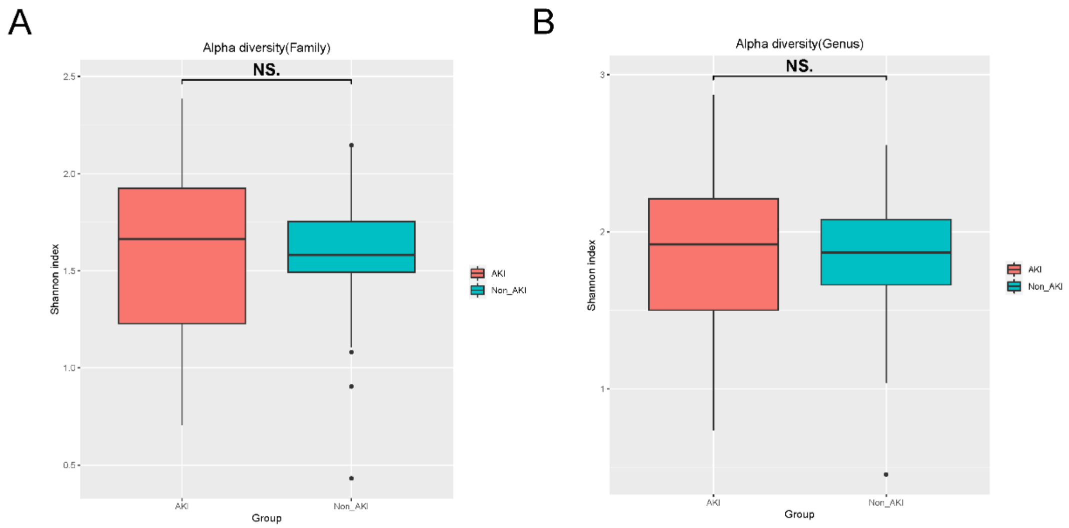

Figure S2. Heatmap shows the association between the differential intestinal microbiota of species and 120 differential pathways (LEfSe) in all patients. Signal asterisks indicate the statistical significance based on Spearman correlation with $P < 0.05$, double asterisks indicate the statistical significance with $P < 0.01$. Cor. coef.: correlation coefficient.

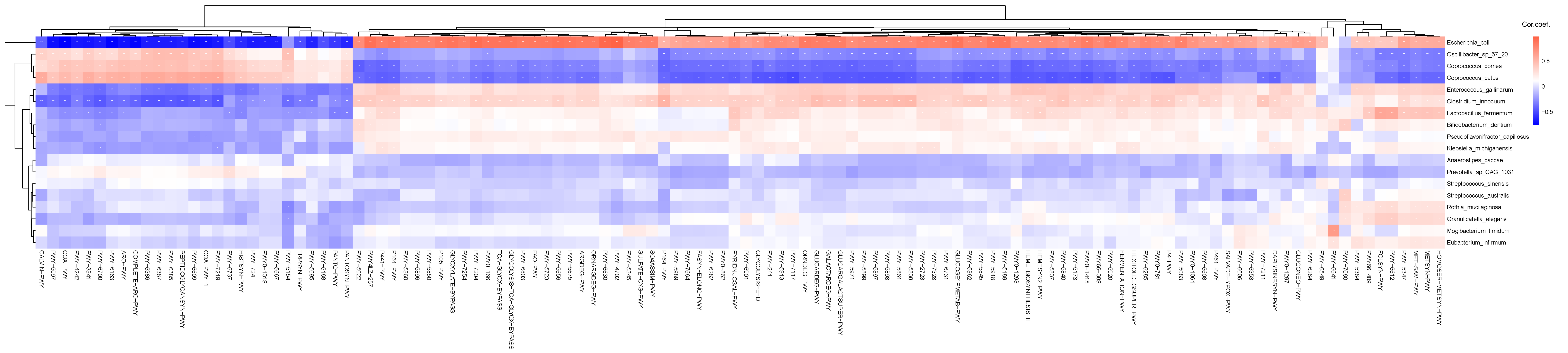

Cor.coef.
0.5
0
−0.5
Escherichia_coli
Oscillibacter_sp_57_20
Coprococcus_comes
Coprococcus_catus
Enterococcus_gallinarum
Clostridium_innocuum
Lactobacillus_fermentum
Bifidobacterium_dentium
Pseudoflavonifractor_capillosus
Klebsiella_michiganensis
Anaerostipes_caccae
Prevotella_sp_CAG_1031
Streptococcus_sinensis
Streptococcus_australis
Rothia_mucilaginosa
Granulicatella_elegans
Mogibacterium_timidum
Eubacterium_infirmum
CALVIN−PWY
PWY−5097
COA−PWY
PWY−4242
PWY−3841
PWY−6700
PWY−6163
ARO−PWY
COMPLETE−ARO−PWY
PWY−6386
PWY−6387
PWY−6385
PEPTIDOGLYCANSYN−PWY
PWY−6609
COA−PWY−1
PWY−7219
PWY−6737
HISTSYN−PWY
PWY−724
PWY0−1319
PWY−5667
PWY−5154
TRPSYN−PWY
PWY−5695
PWY−6168
PANTO−PWY
PANTOSYN−PWY
PWY−5022
PWY4LZ−257
P441−PWY
P161−PWY
PWY−5860
PWY−5896
PWY−5850
P105−PWY
GLYOXYLATE−BYPASS
PWY−7254
PWY−7204
PWY0−166
TCA−GLYOX−BYPASS
GLYCOLYSIS−TCA−GLYOX−BYPASS
PWY−6803
FAO−PWY
PWY−5723
PWY−5656
PWY−5675
ARGDEG−PWY
ORNARGDEG−PWY
PWY−6630
PWY−4702
PWY−5345
SULFATE−CYS−PWY
SO4ASSIM−PWY
P164−PWY
PWY−5989
PWY−7664
FASYN−ELONG−PWY
PWY−6282
PWY0−862
PYRIDNUCSAL−PWY
PWY−6901
GLYCOLYSIS−E−D
PWY−241
PWY−5913
PWY−7117
ORNDEG−PWY
GLUCARDEG−PWY
GALACTARDEG−PWY
GLUCARGALACTSUPER−PWY
PWY−5971
PWY−5899
PWY−5897
PWY−5898
PWY−5861
PWY−5838
PWY−2723
PWY−7328
PWY−6731
GLUCOSE1PMETAB−PWY
PWY−5862
PWY−5845
PWY−5918
PWY−5189
PWY0−1298
HEME−BIOSYNTHESIS−II
HEMESYN2−PWY
PWY−5837
PWY−5840
PWY−5173
PWY0−1415
PWY66−389
PWY−5920
FERMENTATION−PWY
HEXITOLDEGSUPER−PWY
PWY−6285
PWY0−781
P4−PWY
PWY−5083
PWY0−1061
PWY−6608
P461−PWY
SALVADEHYPOX−PWY
PWY−6606
PWY−6353
PWY−7211
DAPLYSINESYN−PWY
PWY0−1297
GLUCONEO−PWY
PWY−6284
PWY−6549
PWY−6641
PWY−7560
PWY−5384
PWY66−409
FOLSYN−PWY
PWY−6612
PWY−5347
MET−SAM−PWY
METSYN−PWY
HOMOSER−METSYN−PWY

Figure S3. Heatmap shows the association between the eight clinical renal functional variables with the abundance of species in the gut microbiome of CSA-AKI patients. Signal asterisks indicate the statistical significance based on Spearman correlation with $P < 0.05$, double asterisks indicate the statistical significance with $P < 0.01$. BUN. pre: preoperative blood urea nitrogen; BUN.d1: blood urea nitrogen on postoperative day 1; BUN.d2: blood urea nitrogen on postoperative day 2; Scr. pre: preoperative blood urea nitrogen; Scr.d1: blood urea nitrogen on postoperative day 1; Scr.d2: blood urea nitrogen on postoperative day 2; Cys-c. pre: preoperative blood urea nitrogen; Cys-c. d1: blood urea nitrogen on postoperative day 1.

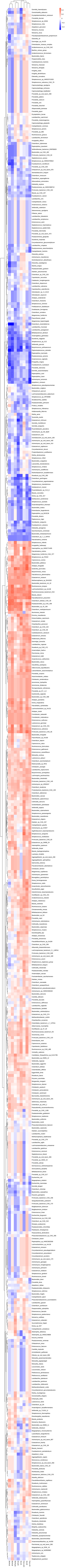

2 Supplementary Tables

Table S1. Relative abundance average of all the family, genus and species levels.

Family level	AKI	Non_AKI
Bacteroidaceae	0.3380	0.3570
Lachnospiraceae	0.1283	0.1347
Ruminococcaceae	0.0972	0.1231
Prevotellaceae	0.0805	0.0847
Enterobacteriaceae	0.0673	0.0496
Eubacteriaceae	0.0238	0.0517
Enterococcaceae	0.0423	0.0272
Tannerellaceae	0.0401	0.0279
Rikenellaceae	0.0268	0.0353
Acidaminococcaceae	0.0099	0.0179
others	0.1459	0.0909
**Genus level**	**AKI**	**Non_AKI**
Bacteroides	0.3439	0.3698
Prevotella	0.0808	0.0872
Faecalibacterium	0.0609	0.0739
Escherichia	0.0588	0.0436
Roseburia	0.0433	0.0401
Eubacterium	0.0242	0.0535
Enterococcus	0.0430	0.0281
Parabacteroides	0.0408	0.0289
Alistipes	0.0272	0.0366
Ruminococcus	0.0177	0.0312
others	0.2594	0.2072
**Species level**	**AKI**	**Non_AKI**
Bacteroides_vulgatus	0.0910	0.1141
Faecalibacterium_prausnitzii	0.0596	0.0710
Prevotella_copri	0.0613	0.0572
Escherichia_coli	0.0574	0.0418
Bacteroides_uniformis	0.0380	0.0460
Bacteroides_plebeius	0.0208	0.0521
Bacteroides_dorei	0.0409	0.0284
Bacteroides_stercoris	0.0275	0.0347
Enterococcus_faecium	0.0325	0.0267
Parabacteroides_distasonis	0.0304	0.0119
Others	0.5406	0.5160

Table S2. Differential pathways in LEfSe analysis.

ID	PATHWAYS	ID	PATHWAYS
FOLSYN-PWY	superpathway of tetrahydrofolate biosynthesis and salvage	PWY-7204	pyridoxal 5'-phosphate salvage II (plants)
PWY-2723	trehalose degradation V	GLUCARDEG-PWY	D-glucarate degradation I
PWY66-389	phytol degradation	PWY0-781	aspartate superpathway
PWY-5154	L-arginine biosynthesis III (via N-acetyl-L-citrulline)	PWY-6353	purine nucleotides degradation II (aerobic)
FAO-PWY	fatty acid β-oxidation I (generic)	PWY-5918	superpathway of heme b biosynthesis from glutamate
PWY-5083	NAD(P)/NADPH interconversion	P105-PWY	TCA cycle IV (2-oxoglutarate decarboxylase)
PWY-5913	partial TCA cycle (obligate autotrophs)	PANTO-PWY	phosphopantothenate biosynthesis I
CALVIN-PWY	Calvin-Benson-Bassham cycle	GLUCARGALACTSUPER-PWY	superpathway of D-glucarate and D-galactarate degradation
P164-PWY	purine nucleobases degradation I (anaerobic)	PWY-5656	mannosylglycerate biosynthesis I
PWY-6385	peptidoglycan biosynthesis III (mycobacteria)	PWY-4702	phytate degradation I
PWY-6606	guanosine nucleotides degradation II	PWY-5384	sucrose degradation IV (sucrose phosphorylase)
PWY-6609	adenine and adenosine salvage III	ARGDEG-PWY	superpathway of L-arginine, putrescine, and 4-aminobutanoate degradation
PWY-6608	guanosine nucleotides degradation III	PWY-5989	stearate biosynthesis II (bacteria and plants)
PWY-6285	superpathway of fatty acids biosynthesis (E. coli)	PWY-5860	superpathway of demethylmenaquinol-6 biosynthesis I
PWY-6282	palmitoleate biosynthesis I (from (5Z)-dodec-5-enoate)	PWY-5861	superpathway of demethylmenaquinol-8 biosynthesis I
GALACTARDEG-PWY	D-galactarate degradation I	TCA-GLYOX-BYPASS	superpathway of glyoxylate bypass and TCA
PWY-6737	starch degradation V	GLYCOLYSIS-TCA-GLYOX-BYPASS	superpathway of glycolysis, pyruvate dehydrogenase, TCA, and glyoxylate bypass
PWY-6731	starch degradation III	PANTOSYN-PWY	superpathway of coenzyme A biosynthesis I (bacteria)
PWY-6549	L-glutamine biosynthesis III	PWY-5723	Rubisco shunt
PWY-5897	superpathway of menaquinol-11 biosynthesis	DAPLYSINESYN-PWY	L-lysine biosynthesis I
PWY-5896	superpathway of menaquinol-10 biosynthesis	PWY-7328	superpathway of UDP-glucose-derived O-antigen building blocks biosynthesis
TRPSYN-PWY	L-tryptophan biosynthesis	PWY0-1298	superpathway of pyrimidine deoxyribonucleosides degradation
PWY-5898	superpathway of menaquinol-12 biosynthesis	P4-PWY	superpathway of L-lysine, L-threonine and L-methionine biosynthesis I
PWY-6901	superpathway of glucose and xylose degradation	PWY-241	C4 photosynthetic carbon assimilation cycle, NADP-ME type
PWY-6641	superpathway of sulfolactate degradation	PWY0-1297	superpathway of purine deoxyribonucleosides degradation
PWY-6163	chorismate biosynthesis from 3-dehydroquinate	PWY-5347	superpathway of L-methionine biosynthesis (transsulfuration)
PWY-6168	flavin biosynthesis III (fungi)	PWY-5345	superpathway of L-methionine biosynthesis (by sulfhydrylation)
ARO-PWY	chorismate biosynthesis I	PWY-7664	oleate biosynthesis IV (anaerobic)
PWY-3841	folate transformations II (plants)	MET-SAM-PWY	superpathway of S-adenosyl-L-methionine biosynthesis
PWY-5920	superpathway of heme b biosynthesis from glycine	PWY-7560	methylerythritol phosphate pathway II
ORNDEG-PWY	superpathway of ornithine degradation	PWY0-1415	superpathway of heme b biosynthesis from uroporphyrinogen-III

GLYCOLYSIS-E-D	superpathway of glycolysis and the Entner-Doudoroff pathway	COA-PWY-1	superpathway of coenzyme A biosynthesis III (mammals)
PWY-7117	C4 photosynthetic carbon assimilation cycle, PEPCK type	PWY-5667	CDP-diacylglycerol biosynthesis I
PWY-5850	superpathway of menaquinol-6 biosynthesis	PWY-5189	tetrapyrrole biosynthesis II (from glycine)
FERMENTATION-PWY	mixed acid fermentation	SO4ASSIM-PWY	assimilatory sulfate reduction I
SULFATE-CYS-PWY	superpathway of sulfate assimilation and cysteine biosynthesis	HEMESYN2-PWY	heme b biosynthesis II (oxygen-independent)
PWY-5022	4-aminobutanoate degradation V	COA-PWY	coenzyme A biosynthesis I (prokaryotic)
PWY-5899	superpathway of menaquinol-13 biosynthesis	P161-PWY	acetylene degradation (anaerobic)
PWY-5862	superpathway of demethylmenaquinol-9 biosynthesis	PWY-7219	adenosine ribonucleotides de novo biosynthesis
PWY-5097	L-lysine biosynthesis VI	PWY-7211	superpathway of pyrimidine deoxyribonucleotides de novo biosynthesis
P461-PWY	hexitol fermentation to lactate, formate, ethanol and acetate	PWY-6612	superpathway of tetrahydrofolate biosynthesis
P441-PWY	superpathway of N-acetylneuraminate degradation	PWY-4242	pantothenate and coenzyme A biosynthesis III
GLUCOSE1PMETAB-PWY	glucose and glucose-1-phosphate degradation	PWY-5971	palmitate biosynthesis (type II fatty acid synthase)
GLYOXYLATE-BYPASS	glyoxylate cycle	COMPLETE-ARO-PWY	superpathway of aromatic amino acid biosynthesis
PWY-6386	UDP-N-acetylmuramoyl-pentapeptide biosynthesis II (lysine-containing)	PWY-7254	TCA cycle VII (acetate-producers)
PWY-6387	UDP-N-acetylmuramoyl-pentapeptide biosynthesis I (meso-diaminopimelate containing)	PWY66-409	superpathway of purine nucleotide salvage
GLUCONEO-PWY	gluconeogenesis I	PWY-6284	superpathway of unsaturated fatty acids biosynthesis (E. coli)
METSYN-PWY	superpathway of L-homoserine and L-methionine biosynthesis	PWY-6803	phosphatidylcholine acyl editing
PWY-6630	superpathway of L-tyrosine biosynthesis	SALVADEHYPOX-PWY	adenosine nucleotides degradation II
PWY0-1061	superpathway of L-alanine biosynthesis	HISTSYN-PWY	L-histidine biosynthesis
PWY0-862	(5Z)-dodecenoate biosynthesis I	PWY-5837	2-carboxy-1,4-naphthoquinol biosynthesis
PWY-6700	queuosine biosynthesis I (de novo)	ORNARGDEG-PWY	superpathway of L-arginine and L-ornithine degradation
PYRIDNUCSAL-PWY	NAD salvage pathway I (PNC VI cycle)	PWY-5838	superpathway of menaquinol-8 biosynthesis I
HOMOSER-METSYN-PWY	L-methionine biosynthesis I	FASYN-ELONG-PWY	fatty acid elongation -- saturated
HEME-BIOSYNTHESIS-II	heme b biosynthesis I (aerobic)	PWY-5675	nitrate reduction V (assimilatory)
PWY-5695	inosine 5'-phosphate degradation	PWY0-166	superpathway of pyrimidine deoxyribonucleotides de novo biosynthesis (E. coli)
PWY0-1319	CDP-diacylglycerol biosynthesis II	PWY-5840	superpathway of menaquinol-7 biosynthesis
PWY-5173	superpathway of acetyl-CoA biosynthesis	PWY-5845	superpathway of menaquinol-9 biosynthesis
PEPTIDOGLYCANSYN-PWY	peptidoglycan biosynthesis I (meso-diaminopimelate containing)	PWY4LZ-257	superpathway of fermentation (Chlamydomonas reinhardtii)
HEXITOLDEGSUPER-PWY	superpathway of hexitol degradation (bacteria)	PWY-724	superpathway of L-lysine, L-threonine and L-methionine biosynthesis II

Table S3. Differential pathways in STAMP analysis.

ID	PATHWAYS	ID	PATHWAYS
ARO-PWY	chorismate biosynthesis I	PWY-5910	superpathway of geranylgeranyldiphosphate biosynthesis I (via mevalonate)
CALVIN-PWY	Calvin-Benson-Bassham cycle	PWY-5913	partial TCA cycle (obligate autotrophs)
COA-PWY	coenzyme A biosynthesis I (prokaryotic)	PWY-5920	superpathway of heme b biosynthesis from glycine
COA-PWY-1	superpathway of coenzyme A biosynthesis III (mammals)	PWY-5971	palmitate biosynthesis (type II fatty acid synthase)
COMPLETE-ARO-PWY	superpathway of aromatic amino acid biosynthesis	PWY-6151	S-adenosyl-L-methionine salvage I
DAPLYSINESYN-PWY	L-lysine biosynthesis I	PWY-6163	chorismate biosynthesis from 3-dehydroquinate
FOLSYN-PWY	superpathway of tetrahydrofolate biosynthesis and salvage	PWY-6168	flavin biosynthesis III (fungi)
GLUCONEO-PWY	gluconeogenesis I	PWY-6282	palmitoleate biosynthesis I (from (5Z)-dodec-5-enoate)
GLUCOSE1PMETAB-PWY	glucose and glucose-1-phosphate degradation	PWY-6284	superpathway of unsaturated fatty acids biosynthesis (E. coli)
GLUDEG-I-PWY	GABA shunt	PWY-6285	superpathway of fatty acids biosynthesis (E. coli)
GLYCOLYSIS-E-D	superpathway of glycolysis and the Entner-Doudoroff pathway	PWY-6353	purine nucleotides degradation II (aerobic)
GLYCOLYSIS-TCA-GLYOX-BYPASS	superpathway of glycolysis, pyruvate dehydrogenase, TCA, and glyoxylate bypass	PWY-6385	peptidoglycan biosynthesis III (mycobacteria)
HEXITOLDEGSUPER-PWY	superpathway of hexitol degradation (bacteria)	PWY-6386	UDP-N-acetylmuramoyl-pentapeptide biosynthesis II (lysine-containing)
HOMOSER-METSYN-PWY	L-methionine biosynthesis I	PWY-6387	UDP-N-acetylmuramoyl-pentapeptide biosynthesis I (meso-diaminopimelate containing)
MET-SAM-PWY	superpathway of S-adenosyl-L-methionine biosynthesis	PWY-6549	L-glutamine biosynthesis III
METSYN-PWY	superpathway of L-homoserine and L-methionine biosynthesis	PWY-6606	guanosine nucleotides degradation II
P164-PWY	purine nucleobases degradation I (anaerobic)	PWY-6609	adenine and adenosine salvage III
P23-PWY	reductive TCA cycle I	PWY-6612	superpathway of tetrahydrofolate biosynthesis
P4-PWY	superpathway of L-lysine, L-threonine and L-methionine biosynthesis I	PWY-6630	superpathway of L-tyrosine biosynthesis
P441-PWY	superpathway of N-acetylneuraminate degradation	PWY-6641	superpathway of sulfolactate degradation
PANTOSYN-PWY	superpathway of coenzyme A biosynthesis I (bacteria)	PWY-6700	queuosine biosynthesis I (de novo)
PEPTIDOGLYCANSYN-PWY	peptidoglycan biosynthesis I (meso-diaminopimelate containing)	PWY-6737	starch degradation V
PWY-241	C4 photosynthetic carbon assimilation cycle, NADP-ME type	PWY-6901	superpathway of glucose and xylose degradation
PWY-2942	L-lysine biosynthesis III	PWY-7117	C4 photosynthetic carbon assimilation cycle, PEPCK type
PWY-3841	folate transformations II (plants)	PWY-7198	pyrimidine deoxyribonucleotides de novo biosynthesis IV
PWY-4242	pantothenate and coenzyme A biosynthesis III	PWY-7210	pyrimidine deoxyribonucleotides biosynthesis from CTP
PWY-5022	4-aminobutanoate degradation V	PWY-7211	superpathway of pyrimidine deoxyribonucleotides de novo biosynthesis
PWY-5097	L-lysine biosynthesis VI	PWY-7219	adenosine ribonucleotides de novo biosynthesis
PWY-5154	L-arginine biosynthesis III (via N-acetyl-L-citrulline)	PWY-724	superpathway of L-lysine, L-threonine and L-methionine biosynthesis II
PWY-5345	superpathway of L-methionine biosynthesis (by sulfhydrylation)	PWY-7384	anaerobic energy metabolism (invertebrates, mitochondrial)

PWY-5347	superpathway of L-methionine biosynthesis (transsulfuration)	PWY-7560	methylerythritol phosphate pathway II
PWY-5392	reductive TCA cycle II	PWY-7616	methanol oxidation to carbon dioxide
PWY-5667	CDP-diacylglycerol biosynthesis I	PWY-922	mevalonate pathway I (eukaryotes and bacteria)
PWY-5695	inosine 5'-phosphate degradation	PWY0-1298	superpathway of pyrimidine deoxyribonucleosides degradation
PWY-5838	superpathway of menaquinol-8 biosynthesis I	PWY0-1319	CDP-diacylglycerol biosynthesis II
PWY-5840	superpathway of menaquinol-7 biosynthesis	PWY0-781	aspartate superpathway
PWY-5845	superpathway of menaquinol-9 biosynthesis	PWY66-367	ketogenesis
PWY-5850	superpathway of menaquinol-6 biosynthesis	PWY66-389	phytol degradation
PWY-5861	superpathway of demethylmenaquinol-8 biosynthesis I	PYRIDNUCSAL-PWY	NAD salvage pathway I (PNC VI cycle)
PWY-5862	superpathway of demethylmenaquinol-9 biosynthesis	SO4ASSIM-PWY	assimilatory sulfate reduction I
PWY-5896	superpathway of menaquinol-10 biosynthesis	SULFATE-CYS-PWY	superpathway of sulfate assimilation and cysteine biosynthesis
PWY-5897	superpathway of menaquinol-11 biosynthesis	TRNA-CHARGING-PWY	tRNA charging
PWY-5898	superpathway of menaquinol-12 biosynthesis	TRPSYN-PWY	L-tryptophan biosynthesis
PWY-5899	superpathway of menaquinol-13 biosynthesis		
